# Phenotypic Analysis of Urothelial Exfoliated Cells in Bladder Cancer via Microfluidic Immunoassays: Sialyl-Tn as a Novel Biomarker in Liquid Biopsies

**DOI:** 10.3389/fonc.2020.01774

**Published:** 2020-09-16

**Authors:** Sandra Carvalho, Catarina M. Abreu, Dylan Ferreira, Luís Lima, José A. Ferreira, Lúcio L. Santos, Ricardo Ribeiro, Vânia Grenha, Mónica Martínez-Fernández, Marta Duenas, Cristian Suárez-Cabrera, Jesus M. Paramio, Lorena Diéguez, Paulo P. Freitas, Marta I. Oliveira

**Affiliations:** ^1^International Iberian Nanotechnology Laboratory, Department of Life Sciences, Braga, Portugal; ^2^Experimental Pathology and Therapeutics Group, Research Center of the Portuguese Institute of Oncology (CI-IPOP), Porto, Portugal; ^3^Porto Comprehensive Cancer Center (P.ccc), Porto, Portugal; ^4^School of Health, Polytechnic Institute of Porto, Porto, Portugal; ^5^Tumor & Microenvironment Group, i3S/INEB, Instituto de Investigação e Inovação em Saúde/Instituto de Engenharia Biomédica, University of Porto, Porto, Portugal; ^6^Faculty of Medicine, Environmental Health Institute, University of Lisbon, Lisbon, Portugal; ^7^Departament of Clinical Pathology, Centro Hospitalar e Universitário de Coimbra, Coimbra, Portugal; ^8^Department of Urology, Centro Hospitalar Do Alto Ave, Guimarães, Portugal; ^9^Genomes and Disease Lab., Research Center of Molecular Medicine and Chronic Diseases (CIMUS), University of Santiago de Compostela, Santiago de Compostela, Spain; ^10^Molecular Oncology Unit, CIEMAT, Madrid, Spain; ^11^CIBERONC, Institute of Biomedical Research, University Hospital “12 de Octubre”, Madrid, Spain; ^12^International Iberian Nanotechnology Laboratory, Department of Nanoelectronics Engineering, Braga, Portugal

**Keywords:** bladder cancer, microfluidics, liquid biopsy, urine, Sialyl-Tn

## Abstract

Bladder cancer is the most common malignancy of the urinary tract, having one of the highest recurrence rates and progression from non-muscle to muscle invasive bladder cancer that commonly leads to metastasis. Cystoscopy and urine cytology are the standard procedures for its detection but have limited clinical sensitivity and specificity. Herein, a microfluidic device, the UriChip, was developed for the enrichment of urothelial exfoliated cells from fresh and frozen urine, based on deformability and size, and the cancer-associated glycan Sialyl-Tn explored as a putative bladder cancer urinary biomarker. Spiking experiments with bladder cancer cell lines showed an isolation efficiency of 53%, while clinical sample analyses revealed retention of cells with various morphologies and sizes. *in situ* immunoassays demonstrated significantly higher number of Sialyl-Tn-positive cells in fresh and frozen voided urine from bladder cancer patients, compared to healthy individuals. Of note, urothelial exfoliated cells from cryopreserved urine sediments were also successfully isolated by the UriChip, and found to express significantly high levels of Sialyl-Tn. Remarkably, Sialyl-Tn expression is correlated with tumor stage and grade. Overall, our findings demonstrate the potential of UriChip and Sialyl-Tn to detect urothelial bladder cancer cells in follow-up and long-term retrospective studies.

## Introduction

Bladder cancer (BC) is the most common malignancy of the urinary tract (1). Cystoscopy and urine cytology are the standard pathological procedures for its detection (2, 3). However, cystoscopy is an invasive and expensive method with limited and operator-dependent sensitivity (4). On the other hand, urine cytology has low sensitivity for low-grade papillary tumors, depends on the examiner's subjective opinion, and displays long turnaround times (5–7). Although most patients are diagnosed with non-muscle invasive BC (NMIBC), which has a 5-year survival rate of 90% (8, 9), high recurrence rates (30–80%) impose long-term cystoscopy and cytology-based follow-ups after transurethral resection of malignant lesions (10, 11). Thus, management of BC is a hurdle with extremely high costs to health care systems (12, 13). To circumvent this issue, distinct BC biomarker assays have been developed targeting tumor-derived proteins or genetic material in voided urine (14–16). A few have reached commercialization, like the Urovysion and Immunocyt kits, showing superior sensitivity when compared to cytology (17). Still, their implementation in clinical diagnosis has been hampered by their high false-positive rates, complexity and high cost (18). In turn, BTA stat and BTA trak tests, which detect urinary human complement factor H-related protein, and NMP22/BladderChek, another protein-based test, have shown to report with limited sensitivity and selectivity for the diagnosis of BC (19–21). Hence, novel platforms and urinary biomarkers that may assist in early detection and monitoring of BC, as a non-invasive and cost-effective strategy, are of outmost importance.

Due to their high throughput and low cost, microfluidic-based diagnostic tools hold the promise for improved patient care and outcomes considering the limitations of current screening and diagnostic techniques as well as the societal and economic impact of BC (22, 23). Microfluidics enables precise manipulation of biological samples and can potentially provide portability and automation, offering exceptional advantages for clinical application (24). A panoply of microfluidic chips have been developed for blood-based biopsies, i.e., isolation of circulating tumor cells (CTCs) and/or tumor-derived material from blood of cancer patients with outstanding results (25–27). In the context of BC, we have recently reported two distinct microfluidic devices for CTC isolation and analysis (28, 29), while Alva et al. evaluated CTCs captured by the commercial system Isoflux (30). Despite the prognostic significance of CTCs in BC, microfluidic analysis of putative cancer biomarkers in voided urine would be ideal, non-invasive and provide significant benefits for patient monitoring, particularly at early stages of the disease, when survival rates are higher and CTCs may not yet be present. Nevertheless, only a scarcity of studies have used microfluidic chips for urine-based BC detection, employing different detection principles, complex systems, and processing times (31–34).

**Figure 1:**
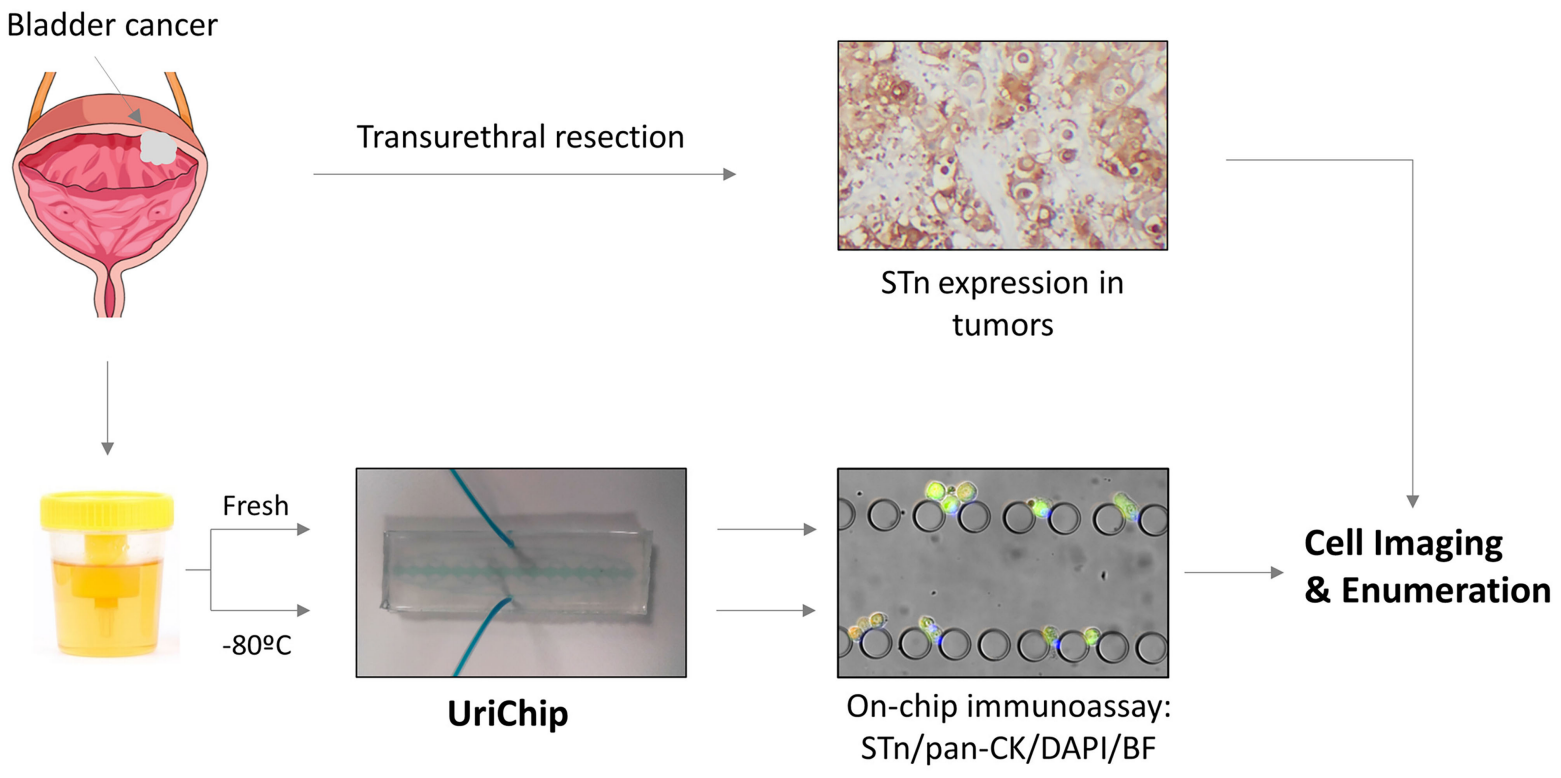
Schematic representation of the enrichment and analysis of urothelial exfoliated cells from bladder washes and voided urine of BC patients using the UriChip. Bladder washes and voided urine from BC patients were collected, subjected or not to cryopreservation and run into the UriChip. A dual biomarker immunoassay was then performed, comprising pan-CK (epithelial marker), STn (tumor associated marker), and DAPI (nuclei staining). Trapped cells were next imaged by immunofluorescence microscopy and nucleated cells expressing STn and/or CK counted. In parallel, corresponding FFPE tissues were screened for STn by immunohistochemistry and results compared to clinicopathological information of patients.

Built on our previous work regarding the development of microfluidic platforms for the capture of BC CTCs (28, 29), we herein report a new microfluidic chip for exfoliated tumor cell (ETC) enrichment from voided urine of BC patients. Importantly, ETCs are valuable sources of information regarding tumor biology and dynamics throughout the course of BC and treatment-follow up (35). A schematic representation of the experimental design used in this study is shown in Figure 1. This label-free strategy, based on cell size and deformability, allowed unbiased retention of ETCs of various sizes and morphologies. Moreover, ETCs were successfully isolated from fresh voided urine samples as well as from cryopreserved urine sediments, which to the best of our knowledge had never been assessed, revealing the feasibility of the system for retrospective analyses. A double marker analysis was performed using pan-cytokeratin (pan-CK) and Sialyl-Tn (STn), a tumor associated antigen overexpressed in BC but absent in the healthy urothelium (36). Our experiments showed, for the first time, STn expression in ETCs from patients, which correlated with staging and grading of BC. Noteworthy, data resulted from two independent data sets, corroborating the versatility of this platform and the potential of STn as a novel biomarker in liquid biopsies.

## Materials and Methods

### Design and Fabrication of the UriChip Microfluidic Device

**Figure 2:**
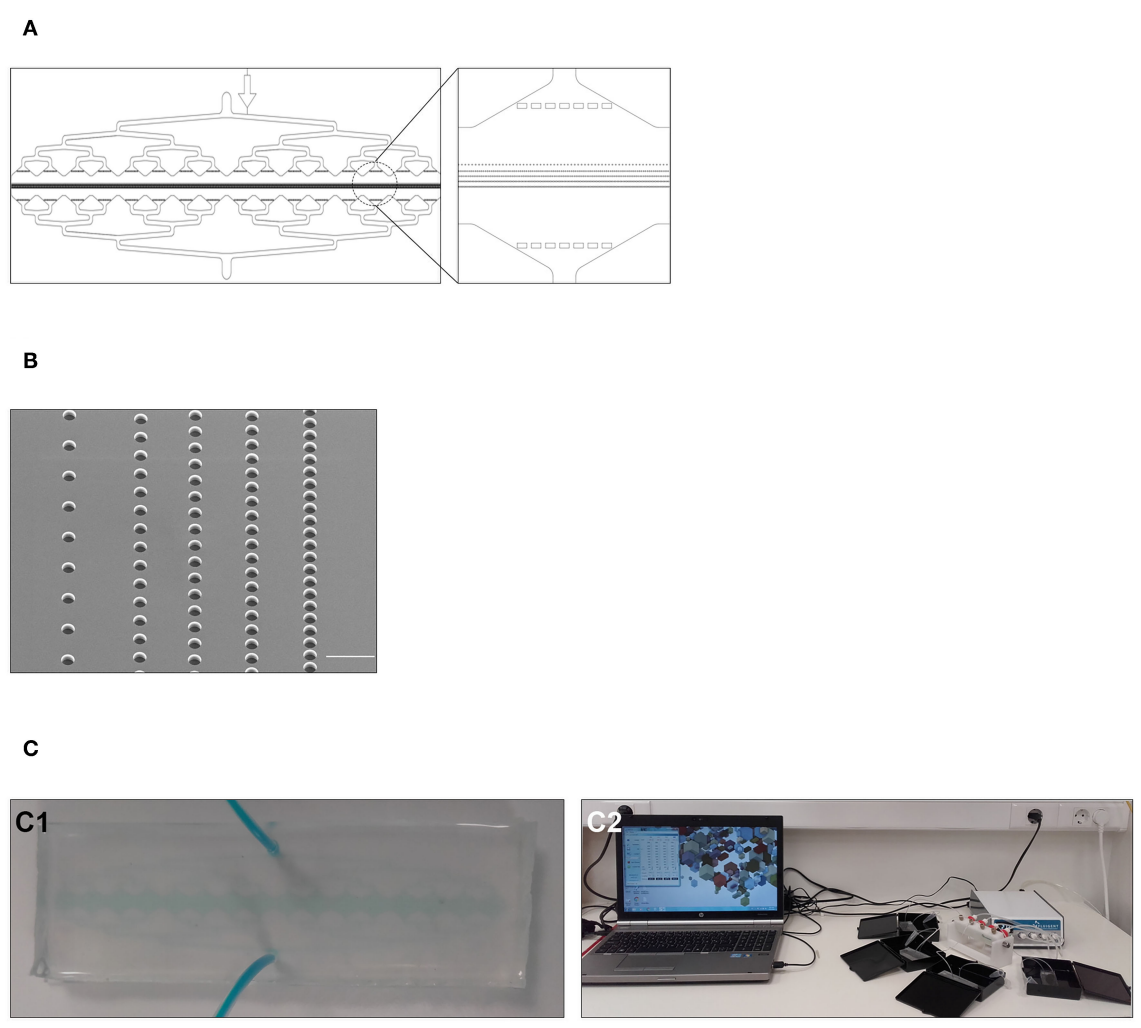
Design and fabrication of the UriChip. **(A)** AutoCAD design of the UriChip. Design includes a circular inlet and outlet, square posts for structural support of the channels, pre-filtering system to prevent debris and large cell clusters, and five row of posts with increasingly narrower gap widths (50, 20, 15, 10, and 5 μm) to separate cells according to their size and deformability. **(B)** Scanning electron microscopy (SEM) image of the five rows of gaps in the master that will give rise to the posts of the UriChip. Scale bar, 100 μm. **(C1)** UriChip after fabrication with PDMS. **(C2)** Experimental setup for simultaneous analysis of four independent samples.

Masters were designed in AutoCAD software (Autodesk, USA) and consist of five rows of posts with increasingly narrower gap widths (50, 20, 15, 10, and 5 μm) (Figures 2A,B), allowing for a wide size-range of urothelial exfoliated cells to be captured (37, 38). A set of square posts with 100 μm gaps were incorporated for structural support of the channels and to prevent the device from clogging with urinary debris and large cell clusters. The design was patterned by direct write laser lithography (DWL 2.0 Heidelberg, Germany) on 200 mm silicon wafers (P/Boron, <100>, Siegert Wafer, Germany). Features with 20 μm depth were defined by silicon deep reactive ion etching (DRIE, STPS Pegasus, United Kingdom) with sulfur hexafluoride (SF6, Sigma 366 Aldrich, USA), and exposed areas passivated with octafluorocyclobutane (C_4_F_8_, Sigma Aldrich, USA). The etching of the features was confirmed by SEM inspection. Photoresist residues were stripped by oxygen plasma (PVA Tepla GIGAbatch 360M, Germany) and the wafer was diced using a DAD 3350 Dicing Saw (Disco, Japan). Masters were then cleaned with isopropyl alcohol (IPA, Sigma- Aldrich, USA), rinsed with deionized water and dried at 150°C. Finally, masters were hydrophobized through treatment with trichloro(1H,1H,2H,2H-perfluorooctyl)silane (97%, Sigma-Aldrich, USA) and cured for 1 h at 65°C.

Devices were fabricated in polydimethylsiloxane (PDMS, Ellsworth Adhesives Iberica, Spain), which was prepared as a two-part system with mix ratio of 10:1 (w/w) base/curing agent, poured over the master, degassed and cured for 2 h at 65°C. Following that, the PDMS was unmolded and inlet and outlet made using a puncher. Irreversible bonding was achieved through surface activation of clean glass slides and PDMS replicas by low power oxygen plasma for 15 s (PDC-002-CE, Harrick Plasma, USA). Immediately after fabrication and bonding (Figure 2C1), microfluidic devices were connected to a multi-channel pressure controller which allows the simultaneous run of up to four independent devices (MFCS™-EZ, Fluigent, France) (Figure 2C2), and channels firstly primed with ethanol 70% (v/v) at 100 mbar to enhance the wettability, then rinsed with 10 mM of Phosphate Buffered Saline (PBS, Sigma-Aldrich, USA) at 200 mbar and lastly treated with 1% (w/v) Pluronic F-127 (Sigma-Aldrich, USA) overnight at 4°C to avoid unspecific cell attachment.

### Cell Culture

Human urinary BC cell lines HT1376 and MCR-STn [overexpressing the sialyl-Tn antigen (39)] were grown in monolayer culture and maintained at 37°C in an atmosphere of 5% CO_2_ in RPMI 1640 GlutaMAX (Invitrogen, USA) and DMEM high glucose (Invitrogen, USA), respectively. Media were supplemented with 10% heat-inactivated fetal bovine serum and 1% penicillin-streptomycin (both from Invitrogen, USA). Cells were continuously monitored by microscopy to ensure they maintained their original morphology. Where appropriate, BC cells were harvested by incubation in 0.25% Trypsin-EDTA (Invitrogen, USA), washed with PBS and labeled with 12.5 μM calcein-AM (Sigma Aldrich, USA) according to the manufacturer's instructions.

### Isolation of Human PBMCs

Peripheral blood (3 mL) was collected from healthy blood donors after informed consent, layered over histopaque-1077 (Sigma-Aldrich, USA) and centrifuged at room temperature for 10 min at 650 g, without active break. Peripheral blood mononuclear cells (PBMCs) were then gently collected from the gradient interface, washed twice and resuspended in PBS supplemented with 2% bovine serum albumin (BSA, Sigma-Aldrich, USA). Cell viability and concentration were determined using the Tuerk solution (Sigma-Aldrich, USA).

### Collection and Processing of Patient Samples

BC patients were enrolled at the Urology department of Hospital da Senhora da Oliveira, Guimarães, Portugal (*n* = 8) and Hospital Universitario 12 de Ocubre, Madrid, Spain (*n* = 6). Voided urine samples (30–50 mL) from 14 patients were collected prior to transurethral resection of bladder tumor (TURBT). Bladder wash samples (10–30 mL) were obtained after flushing the bladder with saline buffer immediately before TURBT. Table 1 summarizes clinicopathological information obtained from the patients' clinical records. Biological samples were processed within 3 h upon collection, being centrifuged at 1,200 rpm for 5 min and washed twice in PBS. Pellets were then resuspended in 500 μL of PBS-2%BSA for immediate microfluidic analysis or frozen at −80°C for later analysis. As a normal control group, voided urine samples (*n* = 6) from healthy subjects were obtained and subjected to the same protocol. Formalin-fixed paraffin-embedded (FFPE) tumor tissue sections were also included in the study. All procedures were performed after patient informed consent and approval by the Ethics Committee of both hospitals.

**Table 1:** Clinicopathological features of patients included in this study.

**Patient variable**	**BC patient (%)**
*N*	14
Invasiveness	
NMIBC	11 (78.57%)
MIBC	3 (21.43%)
Grading	
Low grade	11 (78.57%)
High grade	3 (21.43%)

*NMIBC, non-muscle invasive bladder cancer; MIBC, muscle invasive bladder cancer; BC, bladder cancer*.

### Analysis of Cell Entrapment in the UriChip Devices

HT1376 cells (1,000 cells in 500 μL of PBS) previously labeled with calcein-AM were injected into the UriChip microfluidic devices at two different inlet pressures (200 and 300 mbar) with the help of a pressure pump. Trapped cells were then fixed with 4% (w/v) formaldehyde solution (Sigma-Aldrich, USA) during 20 min and finally washed with PBS. Capture efficiency (CE) of HT1376 cells was determined by imaging and counting the number of calcein-AM-positive cells captured and comparing with the total input.

CE (%) = (captured HT1376 cells)/(total input HT1376 cells) × 100

To determine cancer cell capture purity in the presence of leucocytes (which may be found in the urine due to cancer associated inflammation), calcein-AM-stained HT1376 cells were next spiked in 500 μL of PBS containing unlabeled PBMCs at a 1:10 ratio, run at 200 mbar and fixed with 4% (w/v) formaldehyde solution as described above. Cell purity and PBMC retention were determined according the following formulas:

Purity (%) = [(captured HT1376 cells)/(captured HT1376 cells + captured PBMCs)] × 100

PBMC retention (%) = (captured PBMCs)/(total input PBMCs) × 100

Similarly, to evaluate the efficiency of isolating cancer cells from voided urine, MCR-STn cells were pre-stained with calcein-AM for 30 min at 37°C, spiked in 500 μL of PBS-2%BSA containing urine sediment from healthy subjects and run at 200 mbar. Cells were then fixed with 4% (w/v) formaldehyde solution and finally washed with PBS. The number of calcein-AM-positive MCR-STn cells captured in the device was compared to the total number of MCR-STn cells spiked, and CE determined.

### Immunofluorescence Cell Staining and Detection

Patient samples (processed bladder washes or voided urine) were resuspended in PBS-2%BSA and injected into the chips at 200 mbar. After a 30 min incubation period with no flow to reduce unspecific binding, cells were fixed with 4% (w/v) formaldehyde for 20 min and further treated with 0.25% Triton X-100 in PBS for 5 min to induce cellular permeability. Subsequently, two washes with PBS were performed and mouse monoclonal anti-CK pan-FITC antibody (1:100 Clone C-11) and DAPI (1:1,000, both from Sigma-Aldrich, USA) diluted in PBS-2%BSA were loaded into the UriChips for 1 h in the dark. The immunostaining process ended by washing with PBS before imaging, with the flow rate set at 200 mbar during the entire procedure. Where appropriate, cells were also immunostained using the anti-STn mouse monoclonal antibody clone B72.3 (0.25 μg/mL in PBS-2%BSA, Abcam, UK) for 1 h and 30 min at room temperature, and labeled with a secondary goat anti-mouse IgG-TRITC antibody (1:1,000 in PBS-2%BSA, ThermoFisher Scientific, USA) for one additional hour, prior to cell fixation and permeabilization. Following sample processing, a fluorescence microscopy analysis of the captured cells was performed under an inverted fluorescence microscope (Ti-E, Nikon, Spain). A total of twenty-five fields (20x) per device were selected randomly and the number of captured cells counted. Only DAPI and STn-positive cells with identifiable cellular morphology and well-delimited cytoplasm were considered for cell enumeration, and count normalized to the total number of DAPI-positive cells.

### Tissue Immunohistochemistry

FFPE tissue sections from patients with BC were screened for STn staining using the streptavidin/biotin peroxidase method with the anti-STn antibody, as previously described (29).

### Neuraminidase Treatment

Samples were incubated with *Clostridium perfringens* neuraminidase (0.1 unit/mL, Sigma-Aldrich, USA) for 2 h at 37°C to cleave terminal sialic acid residues from glycoproteins on cell surfaces. The reaction was stopped with PBS washes.

### Statistical Analysis

Statistical analysis was performed using GraphPad Prism, version 5 (GrapPad Software, Inc., La Jola, CA, USA). Data is presented as mean ± SD. Deviation from normality was tested using the D'Agostino and Pearson normality test. The Mann-Whitney test was used for unpaired samples and differences were considered to be significant when *p* < 0.05 (^*^p < 0.05; ^**^p < 0.01; ^***^p < 0.001).

## Results

### UriChip Performance With BC Cell Lines

**Figure 3:**
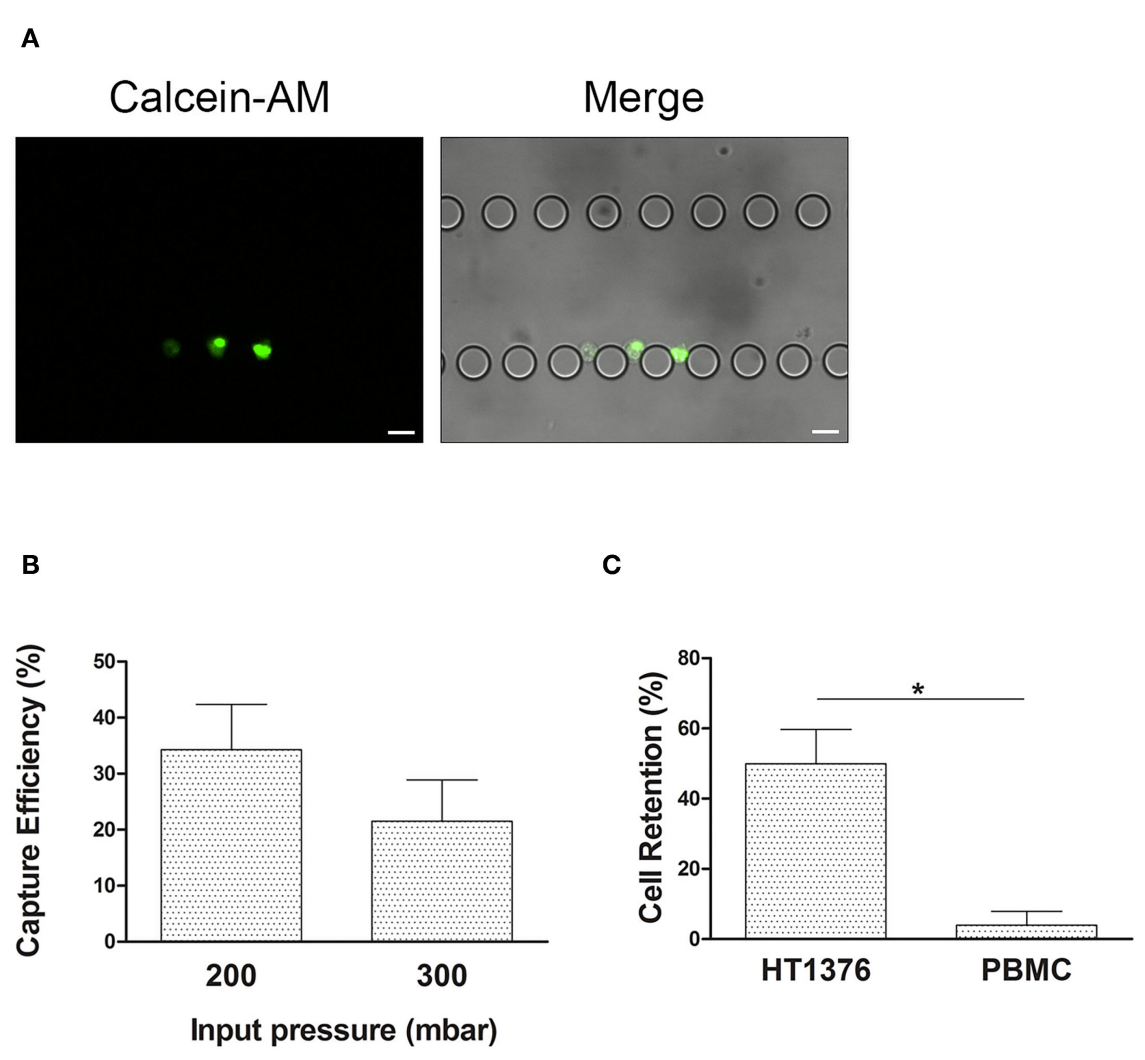
UriChip performance with HT1376 bladder cancer cells. **(A)** HT1376 cells (pre-stained with calcein-AM) captured in the UriChip and visualized by fluorescence microscopy. Scale bar, 20 μm. **(B)** Capture efficiency (CE) of HT1376 cancer cells spiked in PBS and run at 200 and 300 mbar. **(C)** CE of HT1376 cells spiked in PBS solution containing PBMCs at 200 mbar input pressure (50%). Results are described as Mean ± S.D. of three independent experiments. * indicates statistical significance (*p* < 0.05).

The performance of the microfluidic device to capture BC cells was firstly investigated using HT1376 BC cells as a model, pre-stained with calcein-AM and spiked in PBS. As illustrated in Figure 3A, cells captured inside the chip were morphologically intact and remained mostly trapped in the rows of posts with 15–10 μm spacing, in agreement with their average cell size (15.5 μm, Figure S1A), and their higher ability to deform as compared to non-malignant counterparts (40).

To avoid the risk of device leaking and ensure the proper and intact morphology of urothelial exfoliated cells during the microfluidic capture, 300 mbar of inlet pressure was applied in the UriChip. On the other hand, to prevent squeezing of urothelial cells through the microposts under pressure and consequently their loss to the outlet, the minimum inlet pressure of 200 mbar was tested. By working on the range of 200–300 mbar, results revealed a higher CE of HT1376 cells at 200 mbar when compared to 300 mbar input pressure (Figure 3B). This result can be explained by increased hydrodynamic forces acting on the post-trapped cells at higher pressure values, causing cell loss. Hence, in all subsequent experiments the inlet pressure was set to 200 mbar.

Given that the urine of BC patients is heterogeneous and usually contains leucocytes due to cancer associated inflammation, we next investigated the CE of HT1376 cells pre-stained with calcein-AM in the presence of non-labeled PBMCs isolated from healthy donors, spiked in PBS. PBMC population comprises lymphocytes and monocytes that range between 7 and 15 μm in diameter with a high deformability capacity (41). Notably, CE of HT1376 cells increased up to 50% in comparison to single cell population suspensions, while PBMC retention was minimal (Figure 3C). In fact, a PBMC depletion of 96.1% was observed, hence maintaining high sample purity (62%).

### Capture and Analysis of Human Urothelial Exfoliated Cells in the UriChip

**Figure 4:**
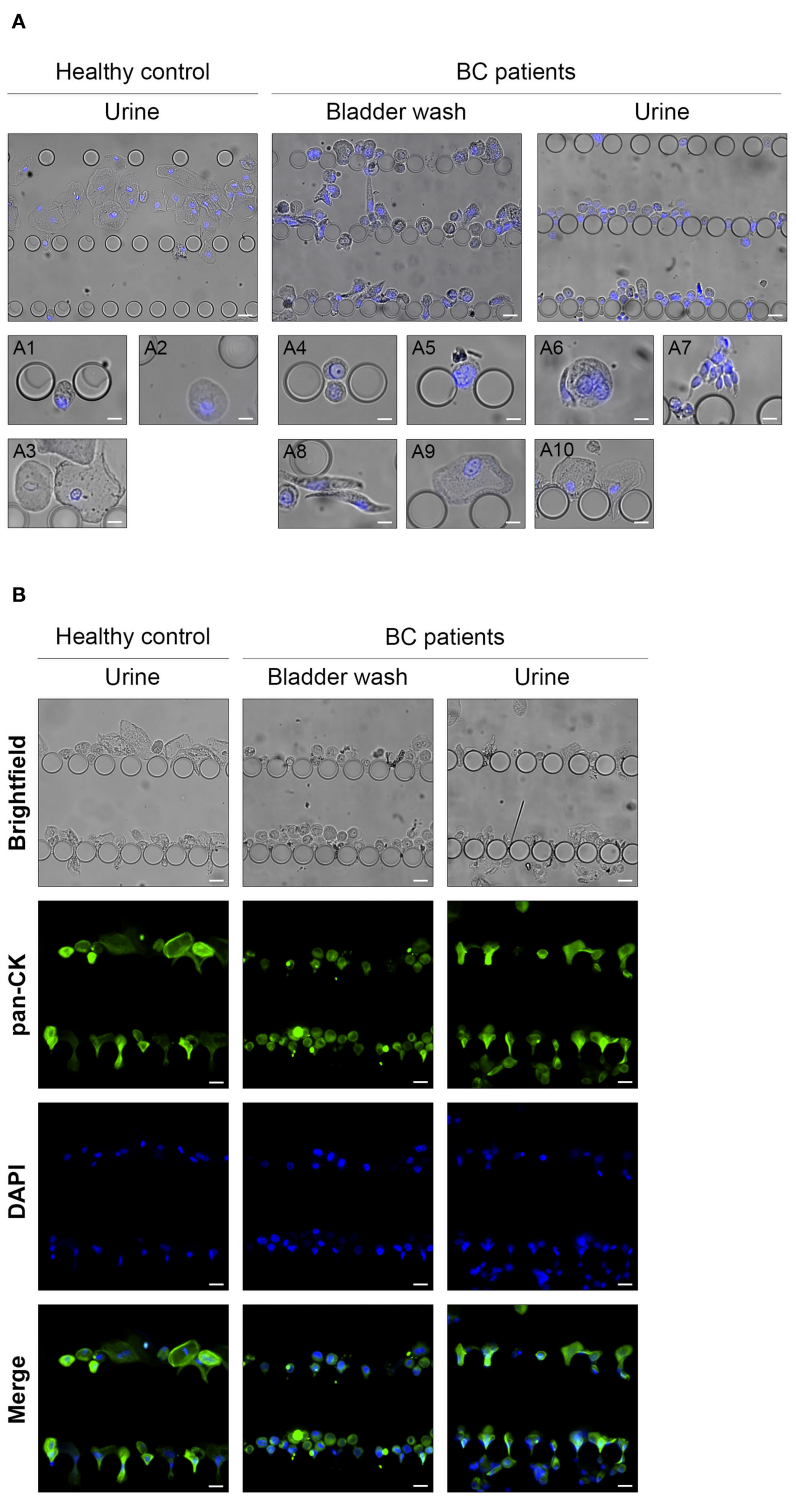
Analysis of UriChip-captured urothelial exfoliated cells from biological samples. **(A)** Microscopy images showing different types of urothelial exfoliated cells from human voided urine and bladder washes retained along the UriChip and stained for the nuclear marker DAPI (blue): (**A1)** basal cell, **(A2)** intermediate cells, **(A3)** umbrella cells, (**A4)** CMV-like cell, **(A5)** cell with a large nucleus, **(A6)** multinucleated cells, **(A7)** cell clusters, **(A8)** spindle-shaped cells, **(A9)** membrane garrulous cells, **(A10)** cell deformability capacity. **(B)** Isolated cells trapped between pillars of the UriChip were stained *in situ* with the anti-pan cytokeratin-FITC antibody (green) and the nuclear marker DAPI (blue). Scale bar, 20 μm.

Having confirmed the ability of the UriChip to isolate BC cells in single and mixed model samples, we further evaluated its potential for the capture and analysis of cells from clinical samples, known to be much more complex and heterogeneous. As such, we analyzed both bladder wash samples, which are highly cellular and contain well-preserved cells (42), as well as voided urine from BC patients and compared them to voided urine from healthy subjects. As expected, several types of urothelial exfoliated cells with distinct morphological features were observed either in healthy controls and patient samples (Figure 4A). Indeed, the urothelium is composed of multiple epithelial cell layers, namely basal, intermediate and umbrella cells (43, 44). Basal cells, which localize on the basal membrane of the bladder lining, are smaller (~10 μm in diameter), mononucleated, and cuboidal-rounded shape (Figure 4A1). Intermediate cells are pyriform in shape, 10–25 μm in diameter, and constitute the majority of the urothelium (Figure 4A2). Umbrella cells, the most superficial cells of the bladder lining, display a very large cytoplasm (25–250 μm in diameter), large and rounded nucleus sometimes bi- or multinucleated, as well as prominent nucleoli (Figure 4A3) (45). In addition, in patient samples, we also identified cells with increased nuclear/cytoplasmic (N/C) ratio, irregular nuclear borders and irregular chromatin patterns. Cells were found either isolated or in clusters, exhibiting various shapes and sizes, including CMV-like atypical cells with an “eye-bird” appearance (Figure 4A4); cells with enlarged nucleus or multinucleated (Figures 4A5,6), clusters of atypical cells (Figure 4A7), spindle-like cells (Figure 4A8), and granular membrane atypical cells (Figure 4A9), as previously reported (46–48). We further evaluated the shape deformation of captured urothelial exfoliated cells as they squeeze through the posts of the UriChip. Large captured cells from healthy controls mostly localized within upper rows of posts (≥20 gap width), while similar sized cells from BC patients were able to deform more and squeeze through narrower posts (Figure 4A10). These observations are supported by the fact that malignant urothelial cells from voided urine present reduced cell stiffness as compared to their healthy counterparts (47).

Cytokeratins are highly expressed in intermediate filaments of normal and neoplastic epithelial cells (49). Hence, to validate the epithelial profile of captured cells from both healthy controls and BC patients, an on-chip immunoassay was performed using a pan-CK-FITC antibody. As illustrated in Figure 4B, the majority of the cells trapped inside the microfluidic device were nucleated and positive for cytokeratin expression, particularly in samples from healthy donors.

These results highlight the potential of the UriChip to retain cells of different sizes and shapes from voided urine, using a label-free approach. Moreover, morphological analysis of captured cells can be performed, and phenotypic characteristics identified via *in situ* immunofluorescence.

### Rigorous Identification of Urothelial ETCs in Clinical Samples

**Figure 5:**
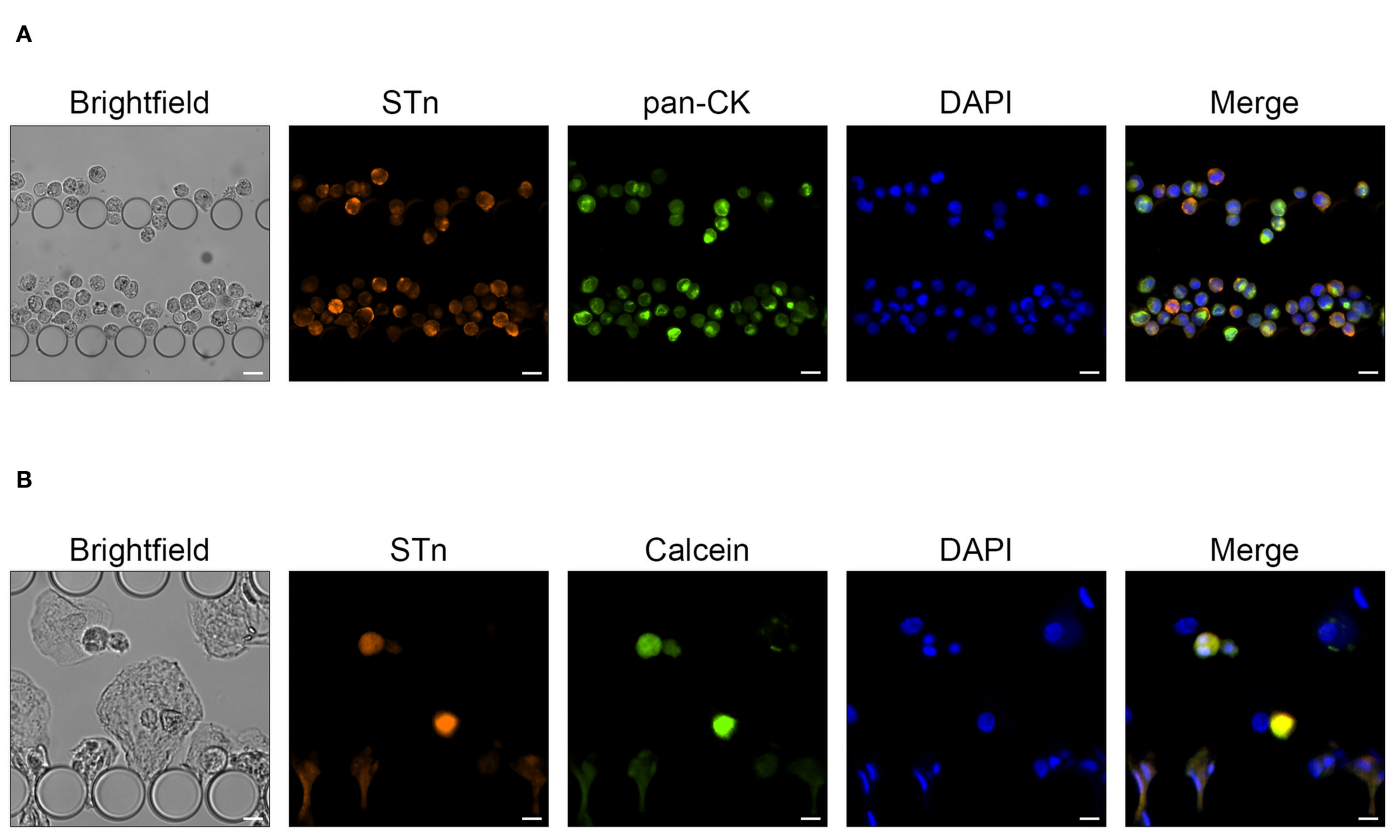
MCR-STn bladder cancer cells captured in the UriChip. **(A)** Immunofluorescence analysis showing the pattern of STn (orange) and pan-CK (green) expression in UriChip-captured MCR-STn cells. STn is mostly expressed at the cell membrane and cytoplasm of cancer cells. Cell nuclei were stained with DAPI (blue). Scale bar, 25 μm. **(B)** MCR-STn cells pre-labeled with calcein-AM (green) spiked in urine from healthy controls. Cell nuclei were stained with DAPI (blue). A capture efficiency of 53% was achieved. Scale bar, 15 μm.

In order to accurately identify urothelial ETCs and distinguish them from benign ones, we selected STn, a tumor associated antigen overexpressed in BC but absent in the healthy urothelium, as malignant biomarker (36). Indeed, STn has been directly associated with BC progression, metastatic potential of neoplastic cells, and decreased overall survival (28, 36). More importantly, we recently reported STn expression in microfluidic-isolated CTCs from BC patients (28, 29). For optimization purposes, we firstly used the invasive BC cell line MCR overexpressing STn antigen (MCR-STn), since STn levels in various non-transduced BC cells lines, including HT1376, are negligible (data not shown) (36). Figure 5A shows morphologically intact MCR-STn cells retained within UriChip and expressing high levels of STn mostly at the cell membrane but also intracellularly. Neuraminidase treatment confirmed STn labeling specificity, since after enzymatic release of STn no signal could be detected in captured cells (Figure S2A). Furthermore, MCR-STn cells were also immunostained for CK and found to be positive, corroborating their epithelial nature and further validating this dual marker microfluidic immunoassay (Figure 5A).

STn expression enhances the migration and invasive capacity of MCR cells (36), which are larger (average cell size of 20 μm) than HT1376 (Figure S1B). In addition, cancer cells become more deformable as they become more invasive. Hence, considering that UriChip captures cells based on their size and deformability, we next reassessed its performance, in even more complex and heterogeneous samples such as urine. For that purpose, MCR-STn cells were pre-labeled with calcein-AM, spiked in voided urine from healthy donors, and stained for STn antigen (Figure 5B). To perform an accurate quantification of the BC cells trapped within the UriChip and rule out possible variations of STn expression levels on MCR-STn cells, the number of calcein and DAPI-positive cells was compared with the total number of MCR-STn cells spiked. Remarkably, the CE was found to be very similar (53%) to that achieved when using HT1376 spiked in PBMCs, demonstrating the good consistency of the UriChip for biological and heterogeneous samples.

### STn Expression of Urothelial Exfoliated Cells Correlates With Tumor Invasiveness and Grade

**Figure 6:**
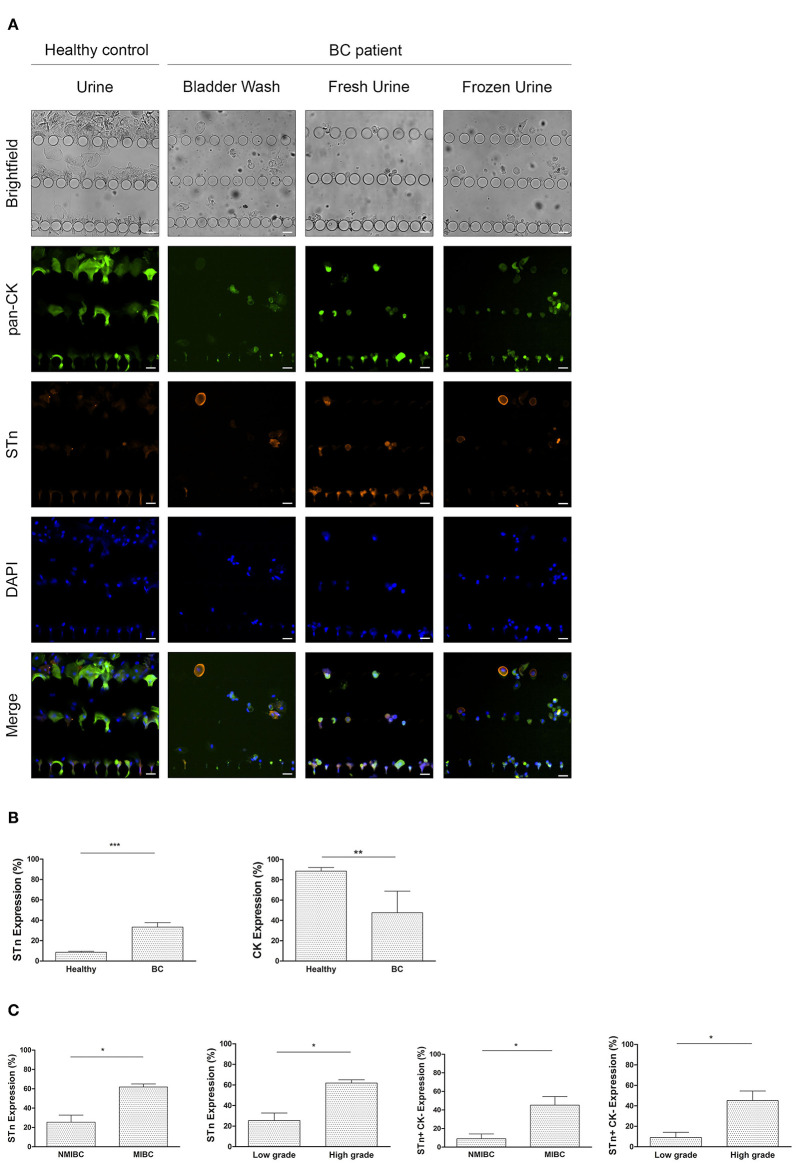
STn expression in bladder urothelial ETC isolated by the UriChip. **(A)** Immunofluorescence staining pattern of urothelial exfoliated cells captured in the UriChip from healthy controls and BC patient samples, namely bladder washes and voided urine, subjected or not to cryopreservation. Cells were probed for STn antigen (orange), pan-CK (green), and DAPI (blue). ETCs were identified according to the following criteria: DAPI-positive, STn-positive, and pan-CK-positive or negative. Scale bar, 20 μm. **(B)** Percentage of STn and pan-CK-positive cells in BC patients vs. healthy controls. As expected, expression of STn is significantly higher in BC (33.28%) compared with healthy controls (8.57%). *p* = 0.0006. In contrast, CK expression decreases significantly in BC (from 8.55% in healthy controls to 47.62% in BC). *p* = 0.0015. **(C)** Correlation of STn-positive and STn-positive/CK-negative cell count with tumor invasiveness and grade. Capture of STn-positive cells and STn-positive/CK-negative cells in the UriChip are significantly higher in muscle invasive BC and high grade bladder tumors (*p* = 0.0127). Statistical significance *p < 0.05, **p < 0.01, ***p < 0.001.

We next moved to the pre-clinical testing of the UriChip using both bladder washes and fresh voided urine from BC patients and a combination of two biomarkers, STn and CK to detect malignant cells. Of note, we extended this analysis and evaluated UriChip and STn/CK immunoassay feasibility to detect cells from cryopreserved samples, greatly relevant for retrospective studies. Figure 6A depicts representative images obtained for each condition tested and shows enhanced STn expression in cells from BC patients as compared to healthy controls. STn labeling was found to be specific as confirmed by the loss of signal upon sample treatment with neuraminidase (Figure S2B). Moreover, STn expression in corresponding tissue sections was evaluated and found to be lower in low-grade NMIBC in comparison to high-grade lesions (Figure S3), while absent in the normal urothelium, in accordance with previous reports (28, 36). CK-positive cells were also found in all samples tested, characteristic of their epithelial origin. Remarkably, results also demonstrated that it was possible to successfully entrap urothelial cells subjected to cryopreservation, which retained the morphologic features and the immunophenotypic markers observed in non-preserved samples (Figure 6A). A quantitative analysis of the captured cells from all fresh and cryopreserved urine samples from BC patients was then performed, revealing that patient urothelial exfoliated cells express significantly higher levels of STn compared to healthy controls (Figure 6B). On the other hand, a significant reduction in the number of CK-positive cells from BC patients was detected (Figure 6B) suggesting a distinct state of differentiation of these epithelial cells. More importantly, we found significant correlations between STn expression and tumor stage and grade, with STn-positive cells decreasing CK expression with higher grade and stage (Figure 6C).

## Discussion

The working principle of UriChip device is based on size-exclusion and cell deformability, which allows for the capture and characterization of urothelial ETCs. In fact, urothelial ETCs, which are shed directly by the growing tumor in the bladder, are known to reflect disease progression (24) and for this reason, they have been widely used as a diagnostic marker in urine cytology. However, the standard diagnostic techniques, are limited considering the requirements for early-cancer diagnosis (1, 50). UriChip has revealed to be a simple and easy-to-use microfluidic device with easy fabrication and cost-effective procedure having the potential to be produced on an industrial scale and thus applied for clinical detection in low-resource settings. Urothelial exfoliated cells of various sizes and intact cellular morphology are efficiently harvested by UriChip from large volumes of patient samples. In addition, the design of five rows of posts with increasingly narrower gap widths enables to handle a wide range of urothelial exfoliated cells, both single and clustered, to be captured and characterized by immunoassays. In addition, the spatially square posts incorporated prevent the built-up of cellular and non-cellular debris and the damage of trapped cells.

Optimization tests were conducted with two distinct BC cell models, prior to the analysis of clinical samples: bladder washes, which contain well-preserved cells when collected before TURBT (42), and voided urine from BC patients, both frequently used for cytological assessment with equivalent results (51, 52). Patient body fluids were then compared to voided urine from healthy subjects. By using a multi-channel pressure controller, which compensates the increase of flow resistance caused by the numerous components of the urine, we were able to efficiently process four independent samples in parallel without rupture or leakage of the system. A few additional studies have also exploited microfluidic chips for urinary cell-based BC detection. However, these involved intricate equipment (31), long incubation periods for surface functionalization and immune affinity selection (34) or lacked clinical validation (32).

Spiking experiments demonstrated that, using the UriChip, the CE of BC cells spiked in leukocytes and urine from healthy donors achieved 53%, typical for size-based filtration (53, 54). Higher efficiencies have recently been reported by Cheng et al., using a size-based microfluidic system, but with less complex model samples, i.e., with BC cells diluted in PBS which do not fully represent the cellular heterogeneity of biological samples (33). Accordingly, cells with different sizes and morphologies were found to be retained in the UriChip, with those originating from BC patients exhibiting higher shape deformation capacity and squeezing though narrower gaps in comparison to similar sized urothelial cells from urine of healthy donors. Consistent with our observations, previous reports using microfluidics and other methodologies confirmed the reduced stiffness of bladder and other cancerous cells compared to their non-malignant counterparts (47, 55–57).

In addition, we phenotypically characterized captured cells *in situ*, via a microfluidic immunoassay involving the epithelial marker, pan-CK, and the tumor-associated glycan, STn. On-chip immunostaining of CTCs isolated from blood samples by microfluidics with distinct biomarkers has been extensively performed (27, 58), and has also been tested with BC urothelial exfoliated cells with good detection accuracy (33), which demonstrates the potential of this approach for effective screening and diagnosis of BC. Based on our previous work showing that more than 90% of BC CTCs captured by a size-based microfluidic device were STn positive (28, 29), we explored the expression of STn in exfoliated cells present in voided urine. Importantly, this antigen is overexpressed in primary bladder tumors, lymph nodes, and distant metastasis, while absent or marginally present on normal urothelium (28, 36), and correlates with decreased overall survival (28). Moreover, STn has been directly associated with a more aggressive phenotype of tumor cells, conferring an invasive potential (59–61). Notably, we found, for the first time, significant high levels of STn in captured urothelial exfoliated cells from voided urine of BC patients. STn expression in corresponding primary tumors was also evaluated in all patients tested and found to be positive. Additionally, STn expression in urinary BC cells significantly correlated with tumor grade and invasiveness, in agreement with STn expression pattern in the tissue, further supporting previously reported findings (28, 29). Notably, a detailed analysis of the STn-positive cell population revealed that these cells display lower levels of CK, indicative of a more mesenchymal-like phenotype. This was particularly evident in more advanced grade tumors, supportive of STn association with tumor aggressiveness. These results suggest that throughout BC progression, urothelial cells undergo an epithelial to mesenchymal transition, with increased potential to invade. Accordingly, we have previously reported that the percentage of CTCs expressing STn was three times higher than those expressing the epithelial marker EpCAM (29), thus linking STn to tumor progression and dissemination.

Parallel to the analysis of fresh clinical samples, we also evaluated cryopreserved patient samples in the UriChip. Results showed that cryopreserved BC cells were successfully isolated and maintained the morphologic features and phenotypic markers, evidencing the versatility of this low-cost system and its feasibility for multi-centric and retrospective analysis on archived samples. To the best of our knowledge, this is the first study evaluating cryopreserved urine sediments by microfluidics. Yet, a large-scale clinical trial is needed to validate the device, particularly for clinical implementation. Proper external regulatory approval would be also necessary. Increasing the cohort of patients with BC (from different grades and stages) will enable to assess the detection accuracy of UriChip. Additionally, further studies by combining UriChip immunoassay with traditional cytology and comparing with commercial available FDA-approved markers are warranted to validate the present findings. Furthermore, the capture and enrichment of intact urothelial exfoliated cells within UriChip provide the opportunity for downstream on-chip proteomic and off-chip single-cell genomic analyses for a precise diagnosis of BC.

Overall, UriChip microfluidic-based platform is able to efficiently capture and enrich urothelial exfoliated cells, from both fresh and frozen voided urine of BC patients, according to their size and deformability. Notably, STn expression in ETCs from patients from two independent data sets was demonstrated herein for the first time, which correlated with staging and grading of BC.

Combination with high-throughput processing and automation may constitute a first step toward a fully integrated system for rapid label-free capture, on-chip phenotypic characterization and enumeration of BC cells. In addition, exploring glycosylation of tumor cells in body fluids, namely STn expression, will offer a more selective malignant cell isolation, paving the way to downstream molecular analysis and fostering precision medicine applications in bladder cancer and other malignancies.

## Data Availability Statement

All datasets generated for this study are included in the article/Supplementary Material.

## Ethics Statement

The studies involving human participants were reviewed and approved by the ethics committee of Hospital Senhora da Oliveira, Guimarães, Portugal. The patients/participants provided their written informed consent to participate in this study.

## Author Contributions

SC was responsible for the study design, patient samples processing, analytical measurements *in vitro*, data analysis, and manuscript writing. CA and LD were responsible for the design and fabrication of the UriChip microfluidic device and evaluation of its performance. DF performed tissue immunohistochemistry experiments. RR, VG, and MD selected and collected patient samples. LL, JF, LS, MM-F, MD, CS-C, JP, and PF contributed to the interpretation of the experimental data. MO was responsible for the project conception, study design, data interpretation, and manuscript writing. All authors discussed the results and commented on the manuscript.

## Conflict of Interest

The authors declare that the research was conducted in the absence of any commercial or financial relationships that could be construed as a potential conflict of interest.
