# Supplementary figures and images for: Phenotypic Analysis of Urothelial Exfoliated Cells in Bladder Cancer via Microfluidic Immunoassays: Sialyl-Tn as a Novel Biomarker in Liquid Biopsies
Source: Front Oncol. 2020 Sep 16;10:1774. doi: 10.3389/fonc.2020.01774 (PMC7526084; doi:10.3389/fonc.2020.01774)

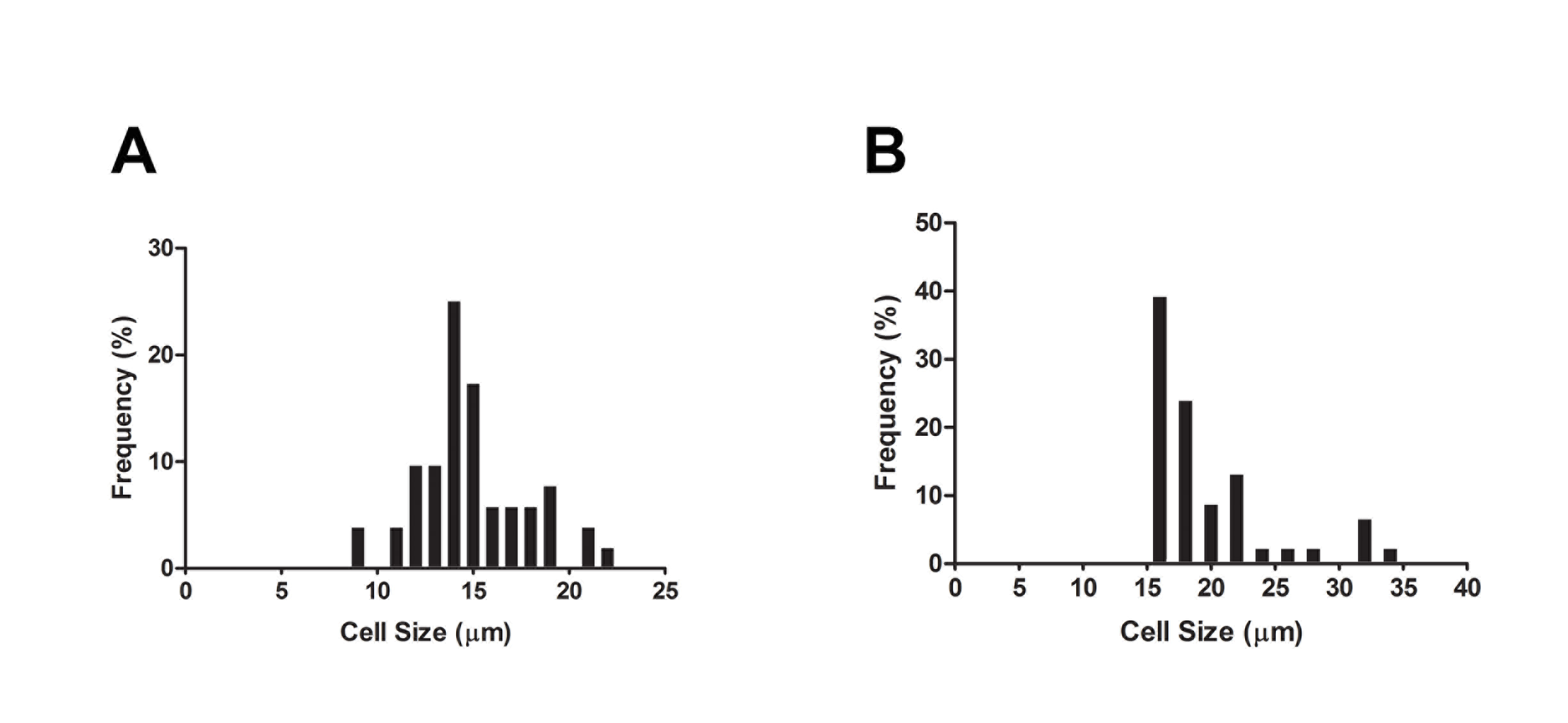

Supplement: Supplementary file 1 [file Image_1.TIF]

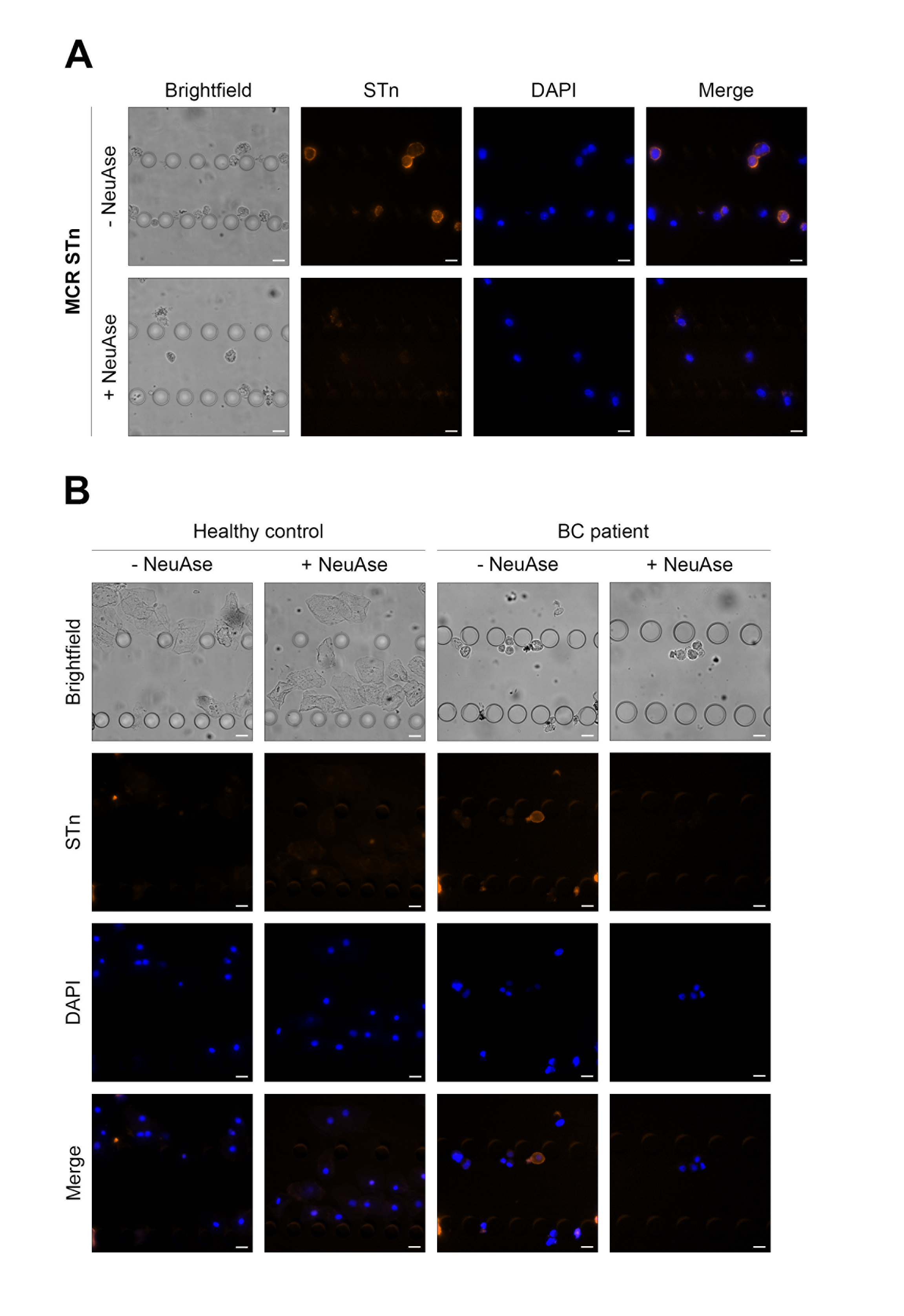

Supplement: Supplementary file 2 [file Image_2.TIF]

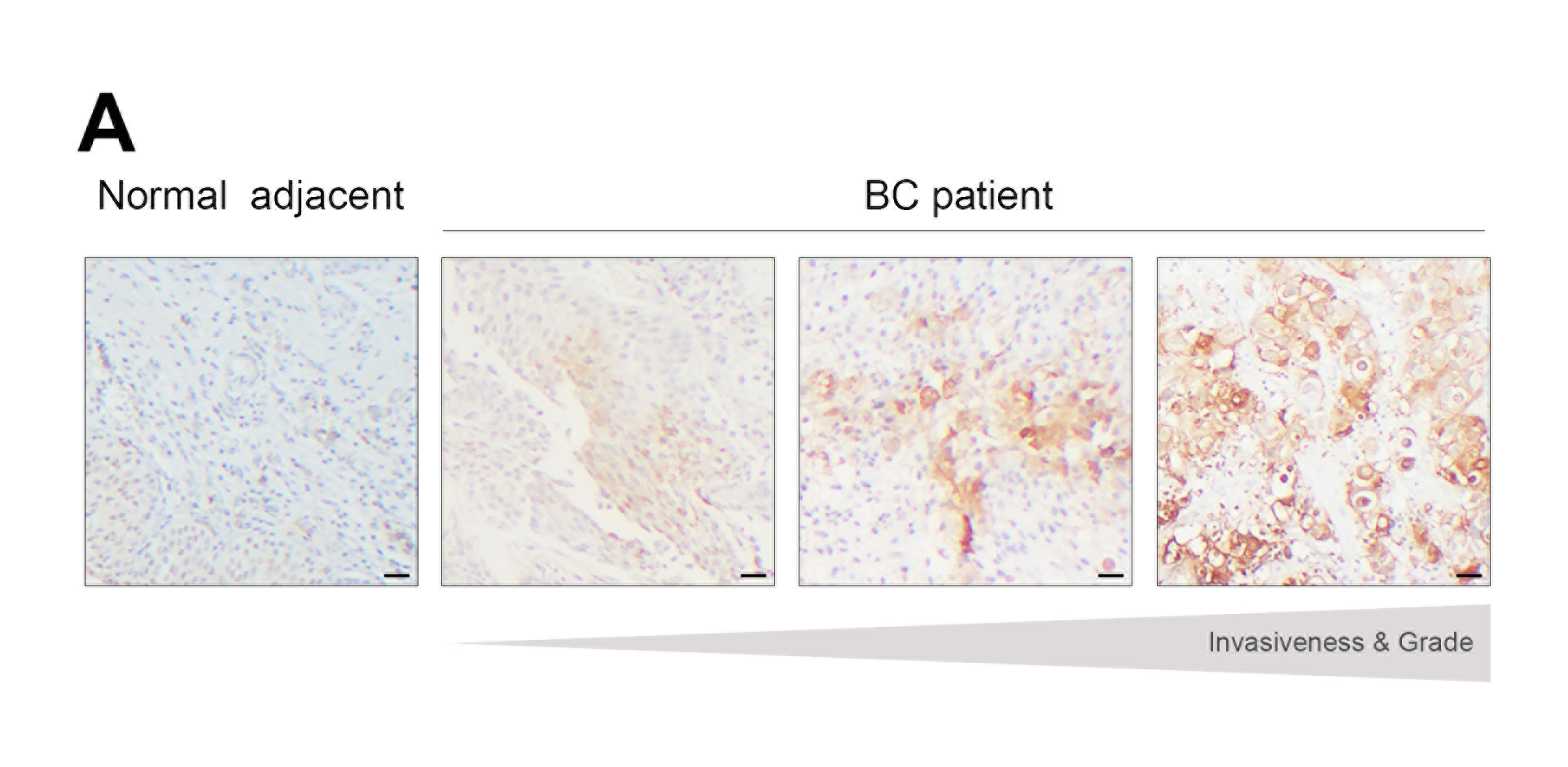

Supplement: Supplementary file 3 [file Image_3.TIF]
